# Oilseed flax cultivation: optimizing phosphorus use for enhanced growth and soil health

**DOI:** 10.3389/fpls.2024.1432875

**Published:** 2024-09-11

**Authors:** Ning He, Fang Huang, Dingyu Luo, Zhiwei Liu, Mingming Han, Zhigang Zhao, Xian Sun

**Affiliations:** ^1^ Yichun Key Laboratory of Functional Agriculture and Ecological Environment, Yichun University, Yichun, China; ^2^ School of Marine Sciences, Zhuhai Key Laboratory of Marine Bioresources and Environment, Guangdong Provincial Key Laboratory of Marine Resources and Coastal Engineering, Pearl River Estuary Marine Ecosystem Research Station, Ministry of Education, Research Center of Ocean Climate, Sun Yat-Sen University, Southern Marine Science and Engineering Guangdong Laboratory (Zhuhai), Zhuhai, China; ^3^ School of Ecology, Sun Yat-sen University, Guangzhou, China; ^4^ Biology Program, School of Distance Education, Universiti Sains Malaysia, Gelugor, Penang, Malaysia

**Keywords:** oilseed flax, phosphorus fertilization, nutrient dynamics, soil bacterial community, soil health

## Abstract

**Introduction:**

Oilseed flax (*Linum usitatissimum* L.) yields are phosphate (P) fertilizer-limited, especially in the temperate semiarid dryland regions of North China. However, there are limited studies on the effects of P-fertilizer inputs on plant growth and soil microorganisms in flax planting systems.

**Methods:**

To address this gap, a field experiment was conducted with four treatments: no P addition and application of 40, 80, and 120 kg P ha^-¹^, respectively. The aim was to investigate the influence of various P fertilizer inputs on yield, plant dry matter, P use efficiency, as well as the population of soil arbuscular mycorrhizal fungi (AMF) and bacteria in dryland oilseed flax.

**Results:**

Our results show that the P addition increased the dry matter, and the yield of oilseed increased by ~200% at 120 kg P ha^-1^ addition with inhibition on the growth of AMF hyphae. The moderate P supply (80 kg ha^-1^) was adequate for promoting P translocation, P use efficiency, and P recovery efficiency. Soil pH, available P, and available K significantly (*p*< 0.05) promoted the abundance of the dominant taxa (*Acidobacteria_GP6, Sphingobacteria* and *Bacteroidetes*). In addition, it is imperative to comprehend the mechanism of interaction between phosphorus-fertilizer inputs and microbiota in oilseed flax soil.

**Discussion:**

This necessitates further research to quantify and optimize the moderate phosphorus supply, regulate soil microbes to ensure high phosphorus utilization, and ultimately establish a sustainable system for oilseed flax cultivation in the local area.

## 1 Introduction

Phosphorus (P) is one of the most limiting nutrients for crops, affecting 30-40% of arable land globally and increasing the demand for P fertilizer (Lang et al., 2018; Yu et al., 2021). The global P fertilizer use has increased from 4.6 million tons in 1961 to approximately 21 million tons in 2015, contributing to the green revolution and food security (Bindraban et al., 2020). However, massive use of P fertilizer has led to water body eutrophication and high P accumulation in soil (Sharpley and Tunney, 2000; Song et al., 2023). The use of a balanced P fertilizer can improve P fertilizer efficiency, as well as soil fertility, which was regarded as an efficient and environmentally friendly method in agricultural production (Guo et al., 2016; Mauchline and Malone, 2017). The economic impact of using phosphate fertilizer in agriculture is substantial, as it significantly affects crop growth, development, and resilience to environmental stresses (Donipati et al., 2023). Phosphorus, a key component of these fertilizers, is essential for energy transfer, photosynthesis, and nutrient movement, leading to higher yields and improved crop quality, ultimately contributing to increased profitability (Zou et al., 2022). Despite cost concerns, particularly for small farmers, the benefits of using phosphate fertilizers often outweigh the expenses (Lambers, 2022). Enhanced yields result in higher marketable produce, boosting farm income. Moreover, phosphate fertilizers enhance crop resilience to drought, pests, and diseases, reducing the risk of crop failure and ensuring stable production (Nelson et al., 2023).

It is essential to prioritize environmental management when it comes to fertilizer use. Excessive application can lead to soil degradation and water pollution, particularly through eutrophication. Thus, it is crucial to emphasize precision agriculture and integrated nutrient management. These strategies are necessary to optimize the use of phosphate fertilizers, ensuring that economic benefits are balanced with environmental sustainability. While phosphate fertilizers may have initial costs, they can yield significant economic returns by enhancing crop quality and improving yields, as long as their application is carefully managed to mitigate environmental harm (Zou et al., 2022). Therefore, effective management of phosphorus in agricultural ecosystems is indispensable for addressing challenges related to food security and environmental degradation.

In soil, P-solubilizing fungi and bacteria constitute approximately 0.1-0.5% and 1-50% of the total population, respectively (Chen et al., 2006; Smith and Read, 2008). The efficiency of crops to absorb P from the soil can be directly and/or indirectly influenced by soil microbes (Richardson and Simpson, 2011; Mbuthia et al., 2015). The microbial abundance, diversity, and composition had various responses to P fertilizer application (Spohn et al., 2015; Tripathi et al., 2018). P-solubilizing microorganisms, including bacteria and fungi, employ various mechanisms to solubilize phosphorus. One of the primary mechanisms is the production of organic acids, such as citric acid, lactic acid, and gluconic acid. These acids lower the soil pH and help dissolve phosphate minerals, releasing soluble phosphorus into the soil solution (Demay et al., 2023). Additionally, these microorganisms produce enzymes like phosphatases that break down organic phosphorus compounds in the soil, converting them into inorganic forms that plants can use (Tan et al., 2013). Another mechanism involves proton extrusion, where some microorganisms extrude protons (H^+^ ions) that aid in dissolving phosphate compounds (Wang et al., 2018). Furthermore, the production of chelating compounds by these microorganisms binds to cations (e.g., calcium, iron, aluminum) associated with phosphorus, freeing the phosphate ions and making them available to plants (Pan and Cai, 2023). P-solubilizing microorganisms are crucial for plant nutrition as they increase the availability of phosphorus in the soil, which is essential for plant growth and development (Sharma et al., 2013). This increased phosphorus availability stimulates root growth and development, enhancing the plant’s ability to uptake water and nutrients (Aberathna et al., 2023). As a result, plants benefit from improved health, increased biomass, and higher crop yields. Moreover, utilizing P-solubilizing microorganisms reduces the need for chemical fertilizers, promoting sustainable agricultural practices and minimizing environmental pollution. By integrating these microorganisms into soil management practices, farmers can achieve more sustainable and productive agricultural systems.

Recent studies have reported that the diversity of the arbuscular mycorrhizal fungi (AMF) community in soils would be reduced by application of P fertilizers (Chen et al., 2014; Lin et al., 2012; Camenzind et al., 2014). Similarly, P fertilization is reported to reduce AMF richness and diversity in plant roots (Liu et al., 2012; Gosling et al., 2013; Liu et al., 2016a). AMF play a vital role in sustainable agriculture by enhancing plant growth and resilience through their involvement in the phosphorus cycle (El-Sawah et al., 2023). AMF form symbiotic relationships with plant roots, facilitating the uptake of phosphorus and other essential nutrients from the soil, which are otherwise inaccessible to plants (Sheteiwy et al., 2023). This symbiosis not only improves nutrient acquisition but also enhances the plant’s tolerance to various abiotic stresses, such as drought and salinity (Nader et al., 2024). By improving soil structure and health, AMF contribute to sustainable agricultural practices, reducing the need for chemical fertilizers and promoting ecological balance (El-Sawah et al., 2023; Nader et al., 2024; Sheteiwy et al., 2023). Their ability to enhance soil key enzyme activities and improve plant growth under stress conditions underscores their importance in achieving sustainable crop production and maintaining soil health.

It is essential to adjust fertilizer schedules to effectively manage the impact of soil microorganisms and optimize agricultural practices (Mander et al., 2012). The availability of phosphorus significantly influences the abundance and diversity of phosphate-solubilizing bacteria, which play a crucial role in mobilizing P into plant-available forms (Li et al., 2023). Long-term P fertilization leads to alterations in soil microbial communities, with high-P soils promoting different bacterial compositions compared to low-P soils (Cheng et al., 2020). Continuous use of P fertilizers can result in an overabundance of certain microbial communities, potentially disrupting soil health (Dincă et al., 2022). Integrating organic matter with inorganic fertilizers can enhance beneficial microbial activity and improve overall soil fertility (Demay et al., 2023). Organic amendments increase the population of bacteria capable of solubilizing inorganic P and contribute to a balanced microbial ecosystem (Demay et al., 2023). Customized fertilization strategies, guided by regular soil testing, should take into account the existing soil P status and microbial community composition to optimize plant growth and maintain microbial balance (Peng et al., 2021). Reducing excessive P application minimizes environmental risks such as runoff and water pollution, promoting sustainable agricultural practices and environmental protection. By implementing these strategies, the impact of soil microorganisms can be effectively managed, leading to improved soil health and sustainable crop production. Therefore, to minimize the impact of soil microorganisms, it may be necessary to make adjustments to fertilizer schedules.

The selection of oilseed flax (*Linum usitatissimum* L.) for this research is based on its economic significance, ability to thrive in semi-arid regions, and its positive impact on soil health. Oilseed flax is a key source of linseed oil, valued for its nutritional advantages and its role in crop rotation (Fao, 2008). Adding phosphorus fertilizer has shown to be effective in increasing oilseed crop yield and enhancing grain quality (Liu et al., 2016b; Powers et al., 2016). However, there is limited knowledge about how varying levels of P-fertilizer affect the growth of oilseed flax and soil microorganisms within the flax planting system, and what the optimal phosphorus application rate is for promoting the growth of oilseed flax and improving soil health. To fill this gap, a field experiment was conducted involving four treatments: application of different P doses and a control group. The goals of this study were to determine: (1) the impact of P fertilizer application on the yield, dry matter, P use efficiency, accumulation, and translocation in oilseed flax, (2) the interaction between soil microbial community composition and structure with varying levels of phosphorus fertilizer inputs and environmental factors, and (3) the economically viable rate of phosphate fertilizer application, in order to contribute towards sustainable agriculture practices and increased productivity.

## 2 Materials and methods

### 2.1 Study site and fertilization treatment

The field experiment is performed at Zhangbei County, Zhangjiakou City, Hebei Province, China (114°57’10” E, 41°7’23” N, altitude 1,430 m). The area experiences a mean annual temperature of 3.2°C, 2300-3100 sunshine hours, 140 KJ cm^-2^ radiation dose, a frost-free period of 90-120 days, annual precipitation of 392.7 mm, and evaporation of 1722 mm. This region is characterized by a semi-arid climate. The soil texture is clay loam. The chemical properties of the topsoil (0~20 cm) before the experiment are shown in **Supplementary Table S1**.

The oilseed flax cultivar in this study was Baxuan 3, which was widely used in the local. A field experiment was set up on May 10, 2017, and harvested on September 24. The experiment included four treatments: a control without P fertilizer, and three different P fertilizer treatments with application of 40 (P40), 80 (P80), and 120 (P120) kg P hm^-1^. The applied mineral fertilizers were urea (N 46%), superphosphate (P_2_O_5_ 16%, Ca 15%), and potassium sulfate (K_2_O 50%). Addition N (90 kg N ha^-1^) and K (90 kg K ha^-1^) fertilizer in all treatments. The experimental design had a completely randomized block design, and the size of each experimental plot was 6 × 10 m, with three replicates. The land management methods employed for the fields adhere to the traditional local model and encompass several specific practices. Soil preparation entails plowing the fields in early spring to a depth of approximately 20-25 cm to loosen the soil and integrate organic matter. For seed sowing, a seeder is used to plant the seeds at a depth of 2-3 cm, with a row spacing of 20 cm to ensure proper plant density and optimal growth conditions. Due to the semi-arid climate, supplementary irrigation is applied during crucial growth stages, particularly during seed germination and flowering, to maintain adequate soil moisture. Weed control is achieved through a combination of manual weeding and the judicious application of herbicides in accordance with local agricultural guidelines. Moreover, pest and disease management involve regular monitoring for pests and diseases, along with the targeted use of pesticides and fungicides as needed to minimize crop damage. Harvesting is performed manually when the majority of the seed capsules turn brown, indicating physiological maturity; the harvested plants are then dried and threshed to extract the seeds. These practices align with regional agricultural methods and optimize the conditions for oilseed flax cultivation in Zhangbei County.

### 2.2 Plant sampling and analysis

The dry weight of the above-ground (shoot) and belowground plant parts (root) and non-grain reproductive parts (including peels, axles, sepals, flower buds, and pedicels) were measured. Samples were collected at key growth stages of oilseed flax: 35 days (budding), 55 days (anthesis), 85 days (kernel formation), and 105 days (maturity). During each sampling, one 1-meter-long plant row was randomly selected from the center of the experimental plot. The root, stem, grains, and non-grain reproductive were separately collected. The **v**arious flax organ samples were isolated and dried at 105°C for 30 min in the thermotank, then dried at 70°C until the weight was constant. The dried samples were thoroughly ground and then filtered with a 1 mm sieve. H_2_SO_4_-H_2_O_2_ decoction and vanadium-molybdenum yellow colorimetry were used to determine the P content in various flax parts (Masoni et al., 2007; Mei et al., 2012). The phosphorus translocation rate (Cassman et al., 1998), contribution rate (Fageria and Baligar, 2003), agronomic utilization rate (Rathke et al., 2006), and recovery efficiency (Rathke et al., 2006) were calculated using the following formulas:


(1) $$\begin{array}{c} \text{Phosphorus translocation rate }\left(\%\right)\\ =\frac{\text{PT}}{\text{P accumulation in organs at flowering stage}}\times 100 \end{array}$$

(2) $$\begin{array}{c} \text{Phosphorus contribution rate }\left(\%\right)\\ =\frac{\text{PT}}{\text{P accumulation in mature seeds}}\times 100 \end{array}$$

(3) $$\begin{array}{c} \text{Phosphate fertilizer agronomic utilization rate}\\ =\frac{\left(\text{crops in the P application area }-\text{ crops in the control area}\right)}{\text{P fertilizer consumption}} \end{array}$$

(4) $$\begin{array}{c} \text{Apparent phosphate recovery efficiency}\;\left(\%\right)\\ =\frac{\text{(P uptake in the above ground part of crops in the P application area - }\text{P uptake of the above ground crops in the control area)}}{\text{P fertilizer consumption}}\times 100 \end{array}$$

Phosphorus translocation (PT, kg ha^-1^) was the accumulation of phosphorus in stems, leaves, and dry matter in the flowering and mature stages and the cumulative amount of phosphorus in dry matter (Masoni et al., 2007).

### 2.3 Soil sampling and preparation processing

Three rhizosphere soil samples were randomly selected from the center rows of the experimental area for each experiment replicate. A hydraulic probe was used to collect the soil core with a depth of 0-200 mm (Giddings Machine Company Inc.), and plastic liners were used to avoid sample contamination. After sampling, soil samples were stored at 4°C till transferred to the laboratory. Then, the mixed soil samples were frozen rapidly to -80°C to preserve their original microbial community and biochemical properties. Soil physicochemical properties were measured through air-dried subsamples.

### 2.4 Soil chemical analysis

A pH meter was used to measure pH value at 1:2.5 (w/v) soil/solution ratio (Thermo ORION STAR A211). A Leco CN-2000 dry combustion analysis meter was used to determine soil organic carbon (SOC) (LecoCorp, USA). Determination of nitrogen (N) in soils was performed with the Kjeldahl method, while the exchange capacity was determined by a new generation of ammonium acetate forced replacement assay. Detection of potassium (K) in soils was carried out using a flame photometer (FP-640, China). Available P (Olsen P) was extracted from soils using 0.5 M sodium bicarbonate (NaHCO_3_) solution. Then, the extracts were subject to colorimetric determination (Olsen, 1954). At each sampling, the alkaline phosphatase (ALP) activities of 1 g wet-weight soil samples were determined (Tabatabai, 1982). P fractionation was performed using the Hedley method (Hedley et al., 1982; Li et al., 2008). 1.0 m HCl-P, 0.1 m NaOH-P, 0.5 m NaHCO_3_-P (pH 8.5), concentrated HCl-P, Resin-P, and residual-P were digested with H_2_SO_4_-HNO_3_. Organic and inorganic P (Pi and Po) were determined using the filtrate derived from 0.5m NaHCO_3_-P (pH 8.5), 0.1m NaOH-P, and concentrated HCl-P fractionation.

### 2.5 AM fungal analysis

Spores of AM fungi in soil were counted using the method described by Daniels (1982). For each sample, spores were extracted from 20 g of soil through a series of sieves. The spores of AM fungi in soil samples were counted on a gridded disk under a binocular stereoscopic microscope at 200 x magnification. The length of the hyphal was determined by Jakobsen et al. (1992). Mycelia lengths were determined by grid line intersection, and AM fungal hyphae and non-AM fungal hyphae were distinguished by irregular septum, binary branching, irregular wall thickness, and/or connection with chlamydia pores (Rillig et al., 2002).

### 2.6 DNA extraction, PCR, and sequencing

Microbial DNA in soil was extracted during the oilseed seedling stage, budding stage, anthesis stage, kernel stage, and maturity stage. The microbial DNA was extracted from a 0.5 g soil sample using the Power Soil DNA Isolation Kit (MO BIO) according to the manufacturer’s protocols (Lopes et al., 2011). The DNA extracts were purified using the Bacteria Genomic Prep Mini Spin Kit (Amersham Biosciences, NJ) and quantified by the Nanodrop-2000 (Thermo Scientific, USA). The DNA extracts were amplified by primers containing the Roche-454 A and B Titanium sequencing adapters, an eight-base barcode sequence in adaptor A, 515 F-5′-GTGCCAGCMGCCGCGGTAA-3 and V4R 5′-TACNVRRGTHTCTAATYC-3′ for the ribosomal region (Wang et al., 2015). The amplicons were quantified by fluorimetry with Pico Green dsDNA quantitation kit (Invitrogen, Life Technologies, Carlsbad, CA). Pyrosequencing libraries were obtained using the 454 Genome Sequencer FLX platform according to standard 454 protocols (Roche 454 Life Sciences, Branford, CT) at Biocant (Cantanhede, Portugal).

All sequences were processed by the UPARSE pipeline, with those at 97% similarity being clustered into operational taxonomic units (OTUs) (Edgar, 2013). Following that, a classification method was assigned to the OTUs by the RDP classifier shipped trained with a 16S rRNA training set (Wang et al., 2007). Samples were resampled to the lowest number of reads (1010) prior to statistical analysis to normalize the sequence read variability among the samples using the rarefy command of the vegan add-on package (Oksanen et al., 2017) in R (3.0.2).

### 2.8 Statistical analysis

ANOVA was performed with SPSS 19.0 after the homogeneity test of multivariate data. The main properties of control oilseed flax and P fertilization were determined by a single degree of freedom control experiment. The significant difference in the measured data was calculated by the T-test (*P* = 0.05). Spearman correlation analysis was used to determine the relationship between microbial gene copy number, plant (biomass, phosphorus concentration, and content) and soil phosphorus variables. A stepwise regression model was built to predict the relationship between soil properties and systemic bacteriology. Prior to permutational multivariate analysis of variance (PERMANOVA), the Mantel test, and redundancy analysis (RDA), rare OTUs (present in less than 10% of the samples in the data set) were removed to reduce inaccurate estimates. Additionally, the changes in α-diversity (including the Simpson and Shannon diversity) of the microbial community due to different treatments were also determined. Heatmaps were used to display the abundance of species in the different samples using the “vegan” package in R (version 4.0.3, http://www.r-project.org/).

## 3 Results

### 3.1 Effects of phosphorus-fertilizer inputs on the phosphorus accumulation

**Figure 1:**
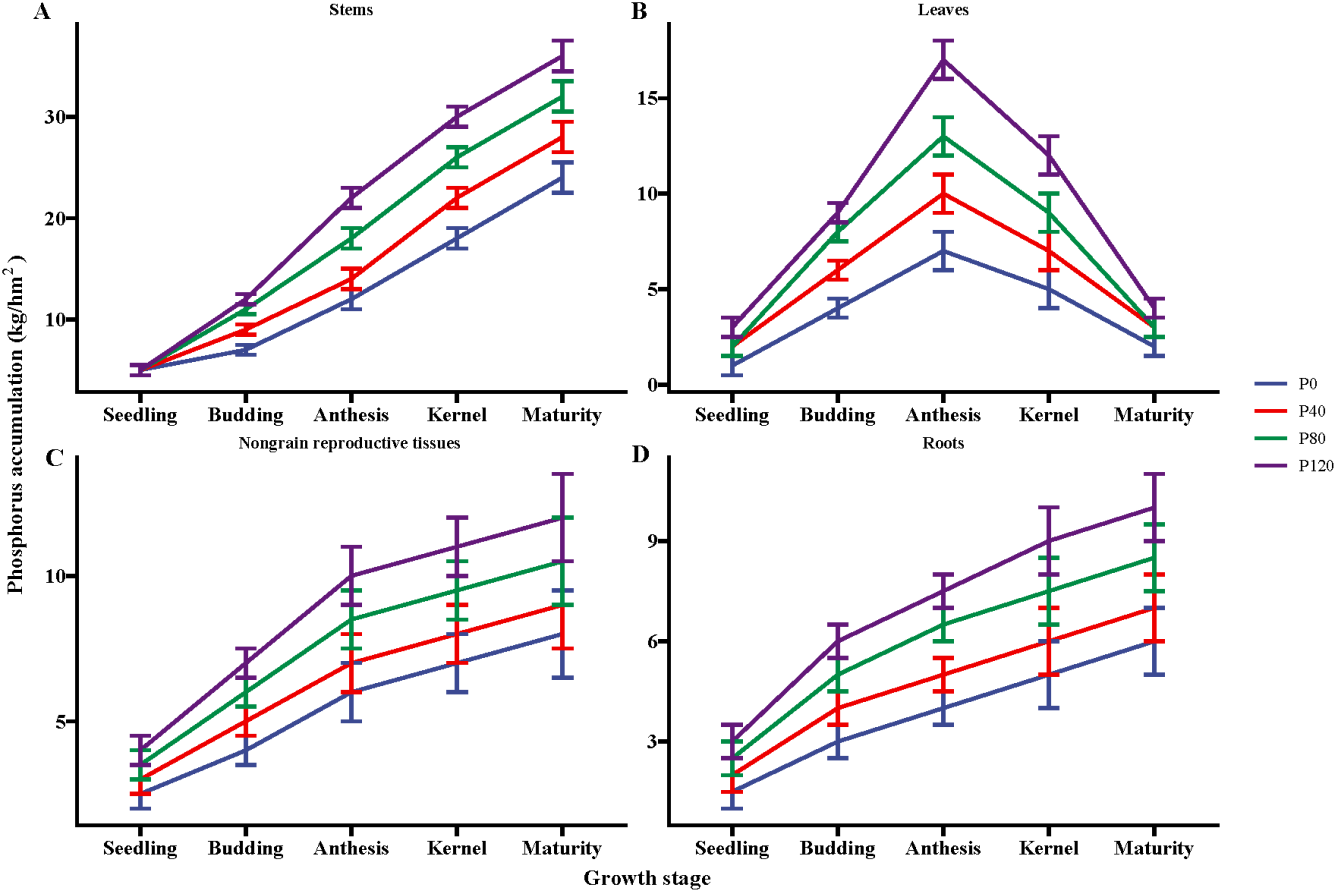
Phosphorus accumulation in the stems **(A)**, leaves **(B)**, nongrain reproductive tissues **(C)**, and roots **(D)** of flax during the growing season affected by different phosphorus management treatments. Bars represent standard error (SE) (n = 4).

P fertilizer inputs significantly increased the P accumulation, as compared with control (**Figure 1**). The P accumulation increased with P addition from 40 to 120 kg ha^-1^ in the different flax organs at five growth stages (seedling, budding, mid-anthesis, kernel-forming, and maturity stage) (**Figure 1**). P accumulation in the leaves reached its maximum at the mid-anthesis stage under the P120 treatment (**Figure 1B**). In contrast, P accumulation in the stems, non-grain reproductive tissues, and roots reached their maximal values at the maturity stage with P120 fertilization. But the P accumulation in leaves was similar between P80 and P120 (**Figure 1B**). In sum, the application of P fertilizer can lead to an increase of about 102% in the total phosphorus contents of the ground aboveground plant parts, including grains (**Figure 1**). The study revealed that the application of phosphorus fertilizer significantly increases phosphorus accumulation in oilseed flax. This enhancement is particularly notable during the mid-anthesis and maturity stages, leading to a substantial overall increase in phosphorus content in the aboveground parts of the plant.

### 3.2 Response of plant dry matter and Olsen-P to phosphorus-fertilizer inputs

P fertilization significantly increased the dry matter of flax, but no significant impact was observed on root/shoot (**Table 1**). Though P fertilizer application increased flax root biomass, no significant difference was found among different P fertilizer treatments (**Table 1**). Olsen-P increased with the increase in P fertilizer application (**Table 1**). Typical P deficiency symptoms were observed in the leaves, roots, no-grains, and stems under nil P treatments in control plots (**Figure 1**; **Table 1**). Olsen-P at P120 showed a higher-level value than P0, P40, and P80, with the maximum value at the anthesis stage (7.03 mg kg^-1^). The maximum ALP appeared at the anthesis stage (**Table 1**). In contrast, the input of P fertilization showed a negligible impact on ALP activity. Compared to control groups, the P concentrations in Resin-P, NaHCO_3_-Pi, NaHCO_3_-Po, HCl-Pi, NaOH-Pi, and residual-P increased significantly at P120 (**Supplementary Table S2**). Phosphorus fertilization significantly increases flax dry matter and soil Olsen-P levels, especially at higher application rates. However, it has little impact on root/shoot ratios, root biomass differences among treatments, and ALP activity.

**Table 1:** Effects of P-fertilizer inputs on biomass, root/shoot ratio, Olsen-P and alkaline phosphatase activities (ALP) in different sampling times.

Sampling time	P-fertilizer input	Dry weight	(g)	Root/shoot ratio	Olsen P (mg kg^-1^)	ALP (nmol g^-1^ soil h^-1^)
		Shoot	Root			
Seedling	P0	6.13b	1.33b	0.22a	0.87c	7.39b
	P40	8.41a	1.71b	0.20a	2.11b	9.31a
	P80	9.96a	2.93a	0.26a	4.32b	10.23a
	P120	10.49a	2.44a	0.21a	8.32a	12.44a
Budding	P0	15.45b	2.43b	0.16a	0.97c	0.63b
	P40	16.41b	2.97a	0.18a	2.23b	9.94a
	P80	17.23a	2.87a	0.17a	4.73a	10.78a
	P120	18.47a	2.46b	0.13b	6.78a	12.48a
Anthesis	P0	21.77b	3.41b	0.16a	1.47d	9.96b
	P40	24.53a	4.69a	0.19a	2.51c	10.13b
	P80	25.69a	5.59a	0.21a	4.89b	11.23a
	P120	25.41a	5.43a	0.21a	7.03a	13.09a
kernel	P0	26.45b	5.73b	0.22a	0.96c	9.31b
	P40	28.99a	6.24a	0.22a	1.78b	9.97a
	P80	32.33a	6.88a	0.21a	4.33a	10.97a
	P120	33.46a	7.03a	0.21a	6.11a	12.95a
Maturity	P0	23.48c	6.47b	0.28a	0.74c	8.43b
	P40	27.71b	6.82a	0.30a	0.23c	8.04b
	P80	29.44a	6.93a	0.25a	3.44b	9.43b
	P120	29.75a	7.11a	0.24a	4.58a	11.23a
Summary of ANOVA						
P-fertilizer input	***	***	***	*	**	*
Time	***	*	**	**	*	*
P-fertilizer input * Time	***	**	**	NS	NS	NS

Significant differences among three phosphorus fertilization inputs and sampling times within each variable are indicated by different lowercase letters. Additionally, *, **, and *** separately represent P<0.05, 0.01, and 0.001, while NS represents not significant.

### 3.3 Response of yield, phosphorus translocation, use efficiency and apparent phosphorus recovery efficiency to phosphorus-fertilizer inputs

The translocation and translocation efficiency of phosphorus in leaves and dry matter presented the highest value at the P80 level (**Table 2**). Similarly, the contribution rate of phosphorus in leaves and dry matter increased with P addition (from 0 to 80 kg ha-1) and then decreased with the increase of P application (> 80 kg ha^-1^). Compared with control groups, the yield of P40, P80, and P120 treatments increased by 229.0, 259.7, and 353.7 kg ha^-1^, respectively (**Table 2**). When the phosphate fertilizer increased by 200% (from 40 to 120 kg ha^-1^), the yield only increased by 1.73% to 7.04%, suggesting the increase in phosphate fertilizer far exceeded the increase in yield. Apparent P recovery (APR) efficiency and agronomic use efficiency of phosphate fertilizer were both increased in oilseed flax exposed to low P fertilization rates (< 80 P kg ha^-1^), with the highest found at the P80 level (**Table 2**). The decrease that followed indicated that the yield-boosting impact of phosphate fertilizer lessens as the input of phosphorus fertilizer increases. The optimal application of phosphorus at 80 kg ha^-^¹ maximizes phosphorus translocation, recovery efficiency, and agronomic use efficiency in oilseed flax. However, higher rates result in diminishing yield returns.

**Table 2:** Effect of phosphorus management treatments on the seed yield, phosphorus translocation, translocation efficiency, contribution rate, agronomic use efficiency, and apparent phosphorus recovery efficiency of oilseed flax.

Treatment	P translocation (kg ha^-1^ Dry weight)		P translocation efficiency (%)		Contribution rate (%)		Seed yield (kg ha^-1^)	Increase seed yield (%)	Agronomic use efficiency (kg kg^-1^)	Apparent P recovery efficiency (%)
	Leaves	Dry matter	Leaves	Dry matter	Leaves	Dry matter				
P0	41.32c	0.21c	37.28c	30.31c	45.31a	4.34c	1543.37b	—	—	—
P40	53.44b	12.45b	46.32b	32.43bc	20.14c	5.01b	1772.36a	20.35	5.13	16.61
P80	66.83a	20.63a	58.38a	46.79a	30.09b	6.44a	1803.11a	25.85	5.23	18.88
P120	59.14b	15.62b	47.31b	34.53b	19.17c	4.39c	1897.11a	32.41	4.76	15.77

The different letters in the same column indicate significant differences among treatments (*P*<0.05).

### 3.4 Effects of P-fertilizer input on the AM fungal diversity, spore density and hyphal length density

The spore density, evenness, Shannon-Wiener diversity index, Simpson diversity index, and AM fungal richness are different at each sampling time (**Table 3**). No consistent model could be used to describe temporal changes in AM fungal diversity indexes. The number of OTUs and diversity indexes did not show any significant difference between P fertilization treatments (**Table 3**). No significant difference in hyphal length density (HLD) was found under different P fertilizer applications, and spore density decreased in the P120 relative to the low P application rate at budding and anthesis stages (**Table 3**). To sum up, sampling time was an essential factor influencing evenness, Shannon-Wiener diversity index, Simpson diversity index, and AM fungal richness. The impact of the P fertilization rate on these indicators was not significant, with the exception of seedling abundance as shown in **Table 3**. Sampling time had a significant influence on AM fungal diversity and spore density. Phosphorus fertilization rates had minimal impact on these metrics, except for a decrease in spore density at higher P levels during specific growth stages.

**Table 3:** AM fungal diversity, spore density and hyphal length density affected by P-fertilizer input and sampling time.

Sampling time	P-fertilizer input	Richness	Shannon-wiener	Simpson	Evenness	Hyphal length density (mg^-1^ soil)	Spore density (g^-^1 soil)
Seedling	P0	8.75a	0.97a	0.21a	0.19a	0.94a	1.77a
	P40	6.75a	0.72b	0.18a	0.09b	0.87b	1.78a
	P80	7.83a	0.83b	0.19a	0.07b	0.82b	1.78a
	P120	8.44a	0.96a	0.22a	0.06b	0.80b	1.77a
Budding	P0	9.42a	0.72a	0.24a	0.17a	0.87a	1.43a
	P40	7.78a	0.33c	0.21a	0.08b	0.83b	1.31b
	P80	8.62a	0.41c	0.23a	0.06b	0.79b	1.27b
	P120	0.14b	0.63b	0.27a	0.05b	0.70c	1.19b
Anthesis	P0	8.47a	0.81a	0.23a	0.14a	0.81a	1.14a
	P40	6.37a	0.45c	0.19a	0.09b	0.83a	1.01a
	P80	6.72a	0.49c	0.21a	0.07b	0.74b	0.97b
	P120	8.71a	0.79a	0.24a	0.06b	0.63c	0.87b
kernel	P0	9.32a	0.94a	0.21a	0.13a	0.71a	1.02a
	P40	7.32a	0.57b	0.17a	0.10a	0.33b	0.71b
	P80	8.21a	0.61b	0.18a	0.08b	0.45b	0.70b
	P120	9.73a	0.87a	0.21a	0.05b	0.40b	0.68b
Maturity	P0	10.12a	1.03a	0.24a	0.17a	0.68a	0.97a
	P40	8.07a	0.71a	0.18a	0.12b	0.57a	0.74b
	P80	9.77a	0.83a	0.20a	0.11b	0.39b	0.61b
	P120	10.03a	0.97a	0.22a	0.10b	0.30b	0.58b
Summary of ANOVA							
P rates		NS	*	NS	NS	NS	*
Time		**	**	NS	**	*	*
P rates * Time		NS	NS	NS	NS	NS	NS

Significant differences among P-fertilizer inputs and sampling times within each variable are indicated by dissimilar lowercase letters according to the Duncan test. Additionally, *, **, and *** separately represent P<0.05, 0.01, and 0.001, while NS represents not significant.

### 3.5 Response of bacterial communities to phosphorus-fertilizer inputs

**Figure 2:**
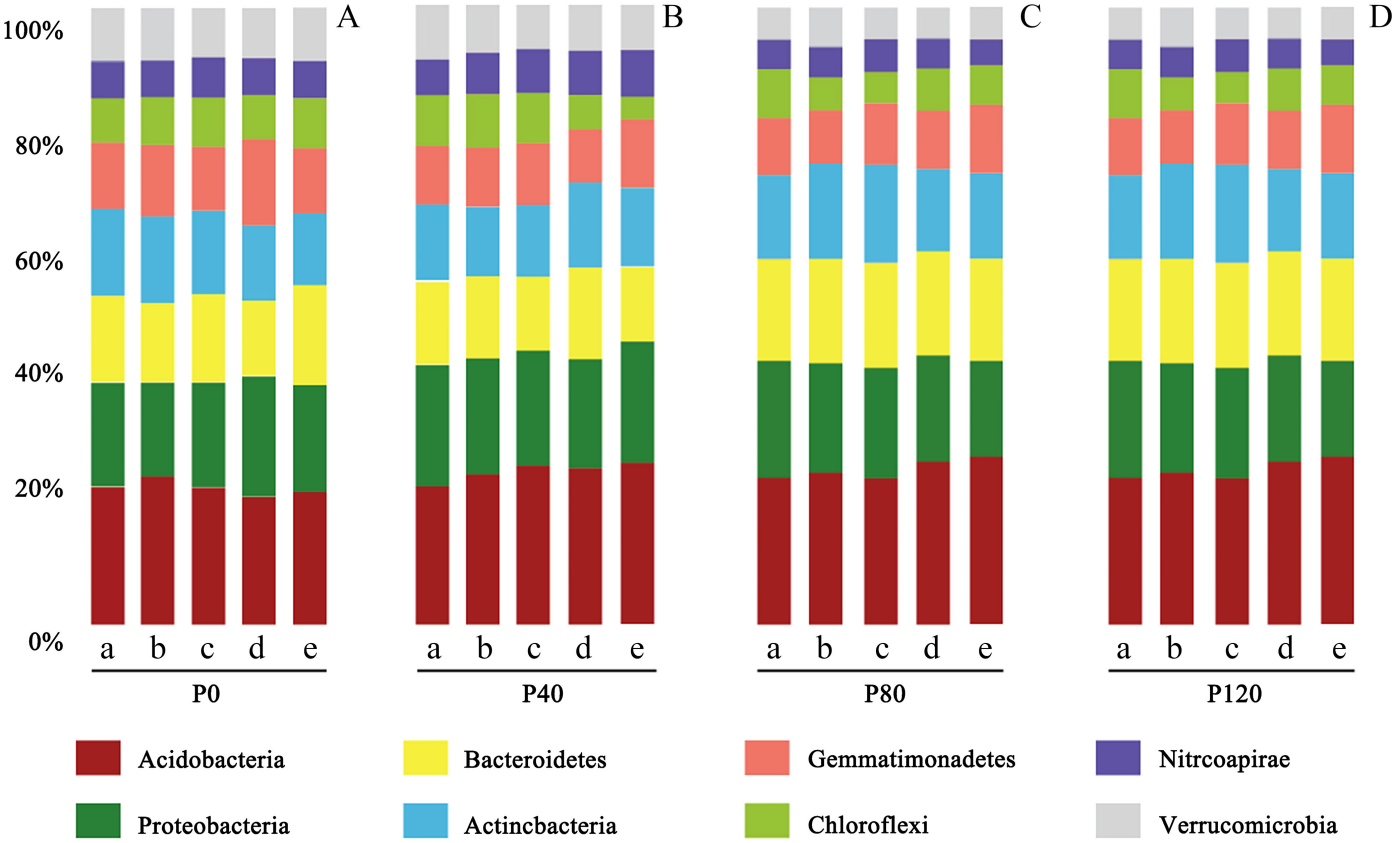
Taxonomic abundance of prokaryotic-16S rRNA OTUs at phylum level at the flax seedling **(a)**, budding **(b)**, anthesis **(c)**, kernel **(d)**, and maturity stage **(e)** influenced by different phosphorus management treatments (**A**: P0, **B**: P40, **C**: P80, **D**: P120). This demonstrates how different levels of phosphorus fertilizer impact the composition of soil bacterial communities. *Acidobacteria* consistently dominate, but increasing phosphorus levels tend to promote the abundance of Proteobacteria and Bacteroidetes.

In this study, according to 16S rRNA gene sequence taxonomy assignments, 28,724 OTUs were obtained from 435,869 soil sequences. The top four phyla were *Acidobacteria* (126,173 sequences, 23.6% of relative abundance), *Proteobacteria* (88,916 sequences, 17.5% of relative abundance), *Bacteroidetes* (66,393 sequences, 15.4% of relative abundance) and *Actinobacteria* (52,910 sequences, 9.43% of relative abundance) (**Figure 2**). It was also consistent with the conclusion of Chu et al. (2010). It indicated that maintaining sufficient soil P can result in higher abundances of *Proteobacteria*. Archaea accounted for less than 5% of the total prokaryote abundance. Most of the archaea species (more than 95%) were classified as the family *Nitrososphaeraceae*.

**Figure 3:**
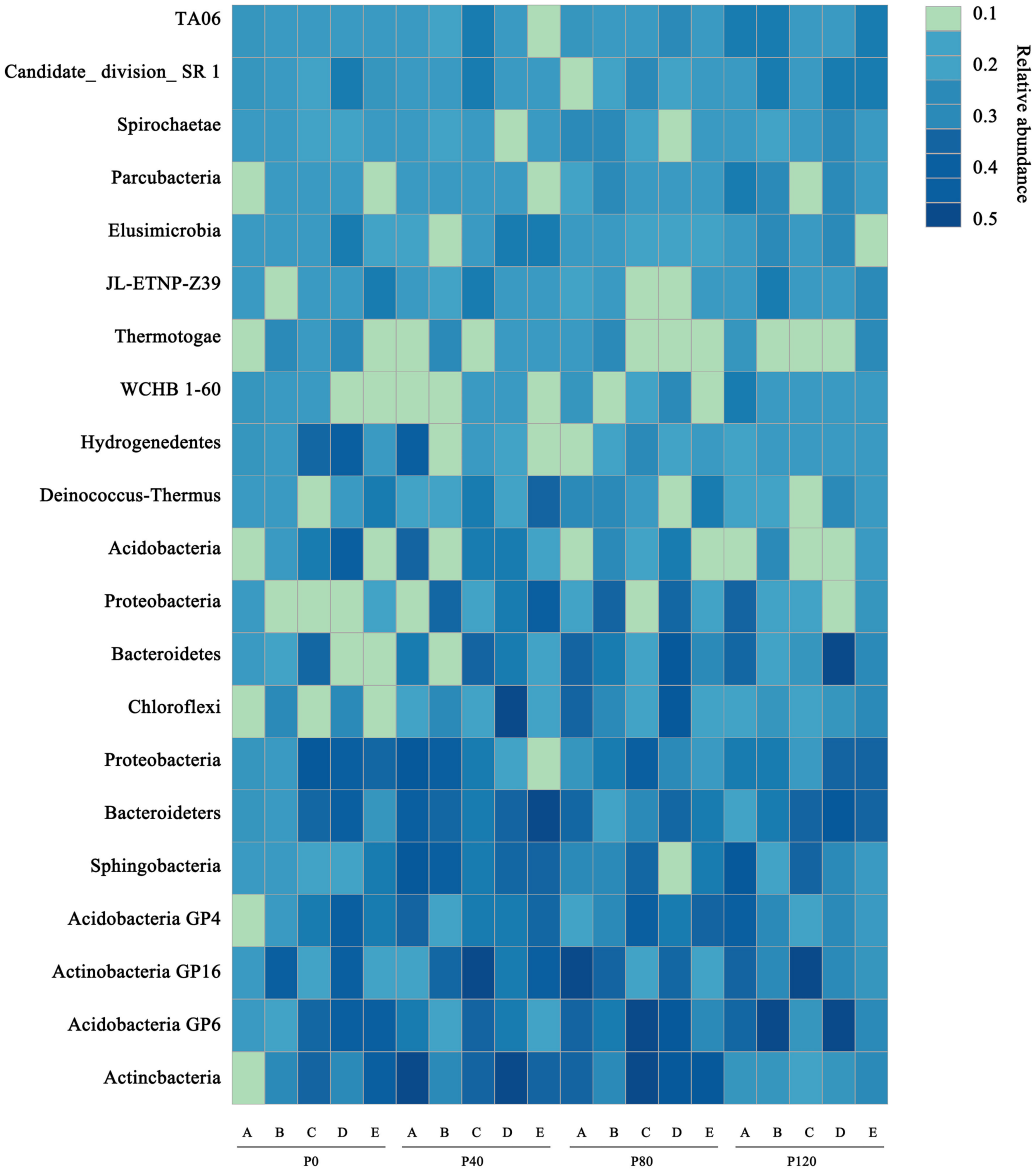
A heatmap showing the dominant classes for flax soils at maturity stage (E) under different phosphorus management treatments. The color scale is rank-based, with the darkest and lightest colors representing the highest and lowest relative abundance of each taxon among the four systems, respectively. The superscript numbers denote marginal (*p* ≈ 0.08) and significant (*P*< 0.05).

Heatmap analysis was used to illustrate the distribution of the seven dominant classes under various P fertilizer inputs (**Figure 3**). P fertilizer input increased the relative abundance of *Bacteroidetes*, *Sphingobacteria*, and Acidobacteria_Gp16 in flax soils, while the abundance of *Proteobacteria* decreased. Furthermore, the abundance of *Bacteroidetes* increased with growth time, and these taxa were the most abundant in P-fertilized soils at the maturity stage. As shown in **Table 4**, P fertilization significantly impacted all classes in flax soils (*P*< 0.05). The combined effect of P fertilizer input and available P significantly impacted *Acidobacteria_GP6*, *Sphingobacteria*, and *Bacteroidetes* in flax soils (*P*< 0.05, **Table 4**). Phosphorus fertilization has a significant impact on the soil bacterial community in oilseed flax fields. It increases the abundance of certain bacterial classes while decreasing others, and this effect is influenced by both P fertilizer input and available phosphorus levels.

**Table 4:** Results from a two-way ANOVA testing the effects of fertilizer regimes (FR), available phosphorus (AP), and FR × AP on relative abundances of the 7 dominant classes in flax soils.

Factors	FR^a^	AP^b^	FR^a^*AP^b^
*Actinobacteria*	5.27**	18.07***	0.94ns
*Acidobacteria GP4*	4.77**	16.82***	0.78ns
*Acidobacteria GP6*	18.23**	16.77***	5.71***
*Acidobacteria GP16*	2.72*	17.88***	1.09ns
*Sphingobacteria*	22.43***	12.72***	2.58*
*Bacteroideters*	12.31***	11.44***	4.78**
*Proteobacteria*	5.11**	14.37***	1.26ns

ns: not significant.

*P< 0.05, **P< 0.01, ***P< 0.001.

a FR: No P fertilization (P0) and P fertilization during flax season.

b AP: different soil available phosphorus before planting flax.

### 3.6 Effects of environmental variables on microbial community

**Figure 4:**
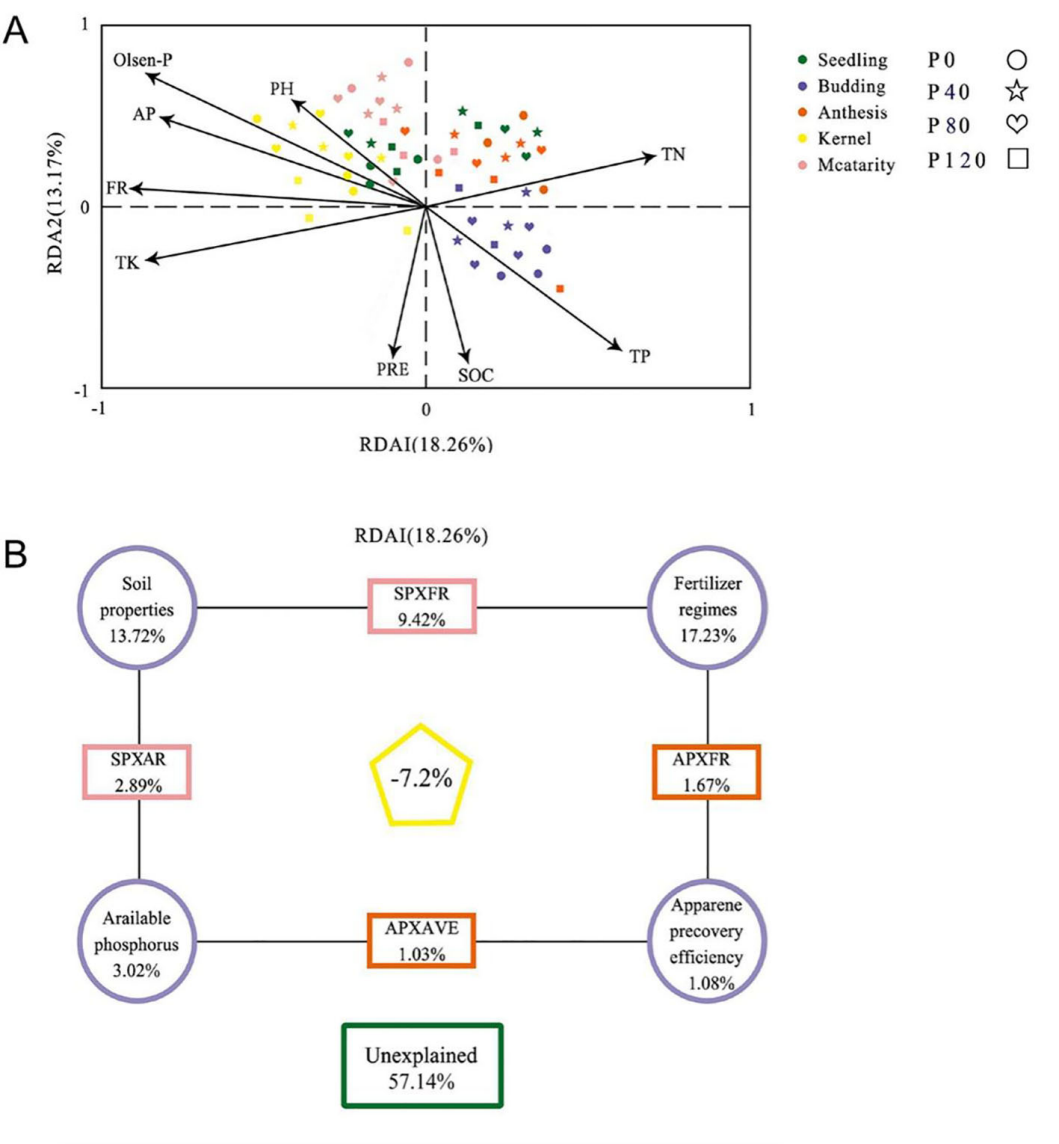
Redundant analysis (RDA) **(A)** and variance distribution analysis (VPA) **(B)** were performed on partial RDA of soil bacterial communities according to available phosphorus (AP) and fertilizer system (FR) from Zhangjiakou City. The RDA factors were selected based on the Variable Expansion Test (VIF). Soil properties include SOC (Soil organic C), TN (total N), TK (total K), apparent P recovery efficiency (AVE), Olsen-P, and pH with different phosphorus management treatments during the 2017 flax season.

The impact of environmental factors on microbial community structure was determined by analysis of variance (VPA) and redundancy analysis (RDA) (**Figure 4**). The first two plots of the RDA accounted for the total data variance of 18.26% and 13.17%, respectively. The results showed a significant correlation between environmental variables and abundant bacterial communities (*P* = 0.002, Monte Carlo test). The combined environmental parameters, P fertilization addition, soil properties, available phosphorus, and P recovery efficiency explained 42.86%, 17.23%, 13.72%, 3.02%, and 1.08% of bacterial community variation, respectively (**Figure 4B**).

The relationship between the dominated bacterial categories and soil properties was evaluated by stepwise regression (**Table 5**). Soil TN and pH accounted for 17.9% and 42.9% of the variations of *Actinobacteria_GP4*, respectively. Soil pH, TN, and TP accounted for 46.1%, 26.7 and 9.1% of the variations of *Acidobacteria_GP6*, respectively. Soil TN, TP, and Olsen-P accounted for 17.5%, 6.8%, and 19.3 of the variations of *Actinobacteria*, respectively. Soil TK, Olsen-P, and SOC accounted for the variations of *Actinobacteria_GP4*. Soil TK, Olsen-P, TN, and TP accounted for 6.9%, 19.9%, 24.6%, and 49.2% of the variations of *Sphingobacteria*, respectively. SOC and TN accounted for 6.7% and 8.8% of the variations of *Bacteroidetes*, respectively. Soil Olsen-P, Tk, and SOC accounted for 29.8%, 16.7%, and 10.8% of the variations of *Proteobacteria*, respectively (**Table 5**). Soil bacterial communities in oilseed flax fields are significantly influenced by environmental variables, especially soil properties and phosphorus management.

**Table 5:** Stepwise regression analysis of the relationship between the relative abundance of 7 dominant classes and soil properties in flax soils.

Classes	Predictor variable	Partial R^2^	Sign	F	*P*
*Actinobacteria*	TN	0.18	+	7.46	0.001
	TP	0.07	–	8.23	0.002
	Olsen-P	0.19	–	13.88	0.001
*Acidobacteria* GP4	pH	0.43	+	41.22	<0.001
	TN	0.18	+	50.46	<0.001
*Acidobacteria* GP6	pH	0.46	+	48.11	<0.001
	TN	0.27	+	56.67	<0.001
	TP	0.09	–	6.88	0.002
*Acidobacteria* GP16	TK	0.32	+	31.07	<0.001
	Olsen-P	0.19	–	8.03	0.005
	SOC	0.10	–	8.13	0.006
*Sphingobacteria*	TP	0.49	–	44.82	<0.001
	TN	0.25	–	18.23	<0.001
	TK	0.07	+	11.22	0.002
	Olsen-P	0.20	+	17.62	<0.001
*Bacteroideters*	TN	0.09	+	4.97	0.005
	SOC	0.07	+	10.23	0.002
*Proteobacteria*	SOC	0.11	–	13.42	0.002
	Olsen-P	0.30	+	21.10	<0.001
	TK	0.17	–	13.80	<0.001

It provides insights into how different soil properties influence the abundance of various bacterial classes in flax soils, which is valuable for understanding soil microbial ecology and managing soil health in agricultural systems. +, significance; -, no significance.

## 4 Discussion

**Figure 5:**
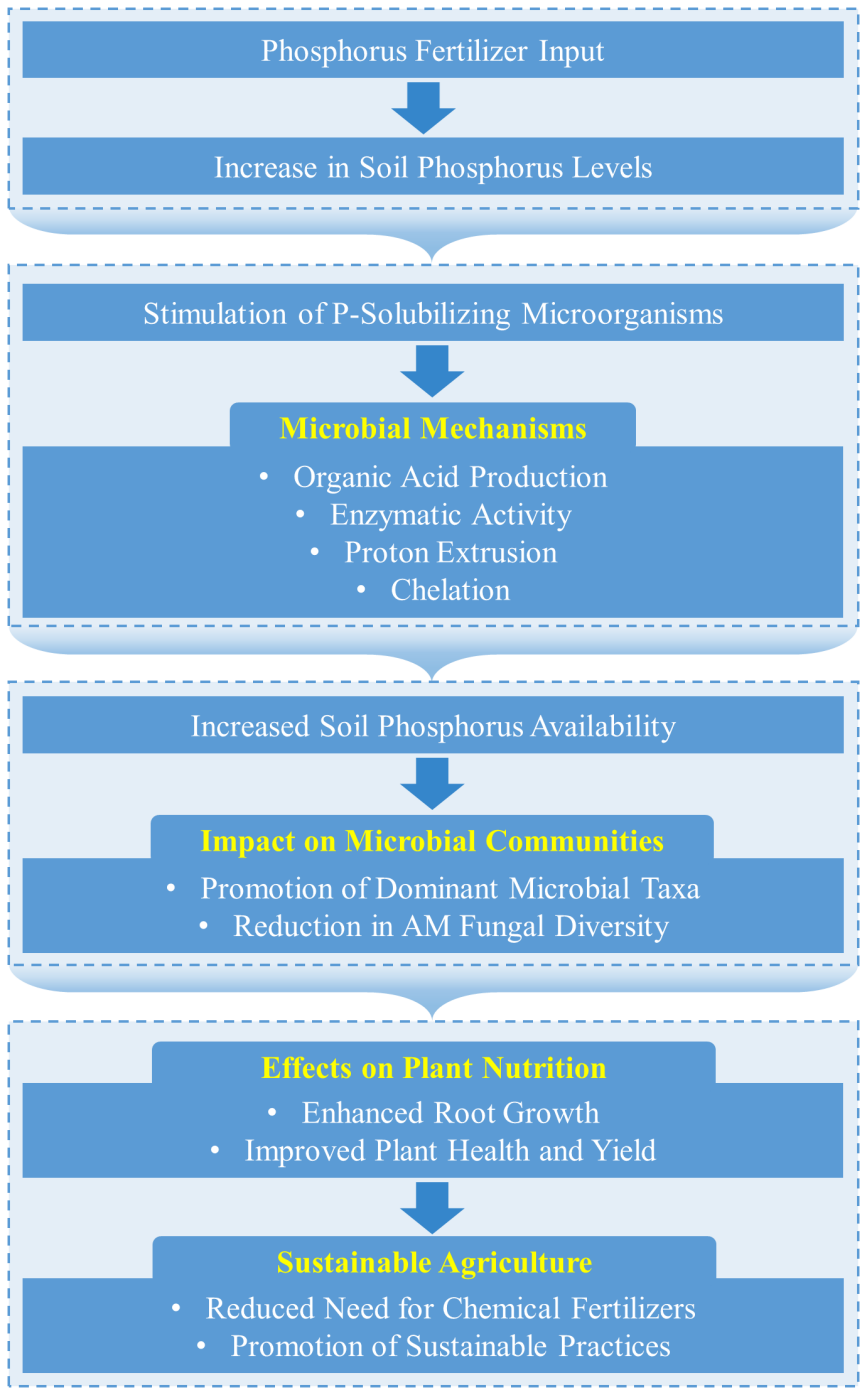
mechanism of interaction between phosphorus-fertilizer inputs and microbiota. Phosphorus fertilizer input initiates a series of beneficial changes in soil chemistry and microbial activity, leading to improved plant nutrition and sustainable agricultural practices. The stimulation of phosphorus-solubilizing microorganisms and their mechanisms play a crucial role in making soil phosphorus more available, which enhances plant growth and reduces the reliance on chemical fertilizers.

The use of phosphorus fertilizer resulted in a substantial increase in phosphorus accumulation from the flowering to maturity stages, leading to a significant shift in phosphorus accumulation in a particular flax organ (**Figure 1**). The enhanced phosphorus accumulation and its redistribution among various plant organs as a result of phosphorus fertilization support the conclusion of the study that optimizing phosphorus application is crucial for improving dry matter accumulation, phosphorus remobilization, and ultimately grain yield in oilseed flax (Xie et al., 2014). The P content in leaves decreased sharply, which inferred that those leaves play an important role in increasing the P content in grains. In wheat, the phosphorus deposited in the seed comes from the vegetative organs and leaves (Peng and Li, 2005). Because the leaves and stems have significant phosphorus translocation efficiency during seed development (Papakosta, 1994; Dordas, 2009). The P translocation efficiency, contribution rate, recovery efficiency, and agronomic use efficiency increase with P addition (from 0 to 80 kg ha-1) but decrease with the increase of P application (> 80 kg ha^-1^) (**Table 2**). Many studies have reported the relationship among yield, yield components, the rate of P fertilizer application, and P accumulation in field and pot experiments (Fageria, 2014; Vandamme et al., 2016). For improved phosphorus acquisition and accumulation in straw biomass, high phosphorus concentration may lead to reduced use efficiency and translocation efficiency for grain production (Wang et al., 2017a). The fiber content in the stem of oilseed flax was higher, suggesting that the stem and non-grain reproductive parts of oilseed flax had a higher demand for phosphorus (Grant et al., 2010), which was consistent with the results of this study. Therefore, a low straw P concentration, which may be beneficial for yield formation, can be used as a criterion for the estimation of high P use efficiency during the selection of genotypes for breeding programs (**Figure 5**).

The input of phosphate fertilizer had a more significant reduction in the diversity indexes of AM fungal (**Table 3**; **Figure 4**), which was consistent with previous research by Zhao et al. (2014). However, the number of OTUs and diversity indexes did not present any significant difference among P fertilization treatments (**Table 3**). These results were consistent with the response of AM fungi community at roots to P input in the exact field location (Wang et al., 2017b). Both shoots and roots will select a stable community of AMF if the same crop has been planted in the same field for a long time. The impact of soil on the diversity of AM fungi may be more significant than its impact on phosphorus availability (Williams et al., 2016; Lang et al., 2018). No significant difference in hyphal length density (HLD) was found under different phosphorus fertilizer applications. Additionally, the spore density of P120 was decreased, relative to the low phosphorus application at the budding and anthesis stages. (**Table 3**). The diversity and growth of AM fungi are significantly impacted by the application of phosphorus fertilizer and the timing of sampling. Optimal diversity and growth of AM fungi are observed at either very low or very high phosphorus levels, depending on the growth stage of maize plants. These findings underscore the importance of phosphorus management in sustaining a healthy AM fungal community, with potential implications for soil health and plant development. These results align with previous studies showing a decrease in spore density (Bhadalung et al., 2005), and HLD did not show any significant change and even decreased under high soil P levels (Lang et al., 2018). Because P enrichment generally suppressed mycorrhizal root colonization and reduced AM fungal hyphae growth (D'Avolio et al., 2014). High P availability in soil reduces C allocation to plant roots, thus directly weakening C-allocation to the AM fungal partner (Johnson et al., 2015). We assume that there should be a P threshold that can act on fungi diversity. More P fertilization gradient experiments need to be set in the future to verify our hypothesis.

It is well established that soil pH is the most vital factor in structuring bacterial communities (Rousk et al., 2010; Shen et al., 2013). We detected that the relative abundance of *Acidobacteria* was significantly affected by soil pH (**Figure 4**, **Table 5**); which agreed with the recent findings by Shen et al. (2013) and Wang et al. (2018). Moreover, it was also found that other soil properties like soil TN were positively correlated with the community composition of Acidobacteria_GP4 and Acidobacteria_GP6, which are consistent with the findings of Nacke et al. (2011) and Zhao et al. (2014). Soil properties, especially TP and Olsen-P, were the main factors that influenced the dominant bacterial classes in the soil. The relative abundance of *Acidobacteria* was significantly correlated with soil TN, TP, and Olsen-P, suggesting that *Acidobacteria* thrives in soils when available P increases Wang et al. (2017). *Bacteroidetes* are highly correlated with TN and SOC in the soil, which is consistent with the previous study of Nacke et al. (2011). However, this study showed that there was no significant effect of P fertilization on the relative abundance of Acidobacteria or Proteobacteria despite the fact that Olsen-P was higher in treatments than in control (**Table 1**; **Figure 2**), which was a discrepancy with Fierer et al. (2005) and Campbell et al. (2010). Though soil microbial community variation is related to multiple indicators even under P fertilization, the supplement and limitation of P may be an essential factor for affecting the microbial community.

## 5 Conclusions

To sum up, the moderate-P supply (80 kg ha^-1^) not only promoted the productivity of oilseed flax but also was good for increased P translocation and P use efficiency. High P-fertilizer input has no significant effects on fungal diversity, and it even backfired. However, phosphate fertilizer action is a primary driving factor of the structure of bacteria in the soil planted with flax. So far, phosphorus management in such soil types has not exerted any negative influence on microbial communities in the soil. The accumulated evidence highlights the importance of incorporating microbial communities to maximize the absorption efficiency of phosphorus. Therefore, it is crucial to understand the interaction between flax and microbiota, use soil microbes most effectively through a fertilization regime, and finally create a sustainable system for flax cultivation in local areas. In the future, the interaction between P cycling and microbial function, as well as the specific mechanism of influence on plant growth by P fertilizer, should be fully explored to design and develop more efficient agricultural management schemes.

## Data Availability

The original contributions presented in the study are included in the article/**Supplementary Material**, further inquiries can be directed to the corresponding author/s.

## References

[B1] Aberathna A. Satharasinghe D. Jayasooriya A. Jinadasa R. Manopriya S. Jayaweera A.. (2023). Managing Soil and Plant Nutrients: Role of Microbial Phosphate Solubilisation.

[B2] Bhadalung N. N. Suwanarit A. Dell B. Nopamornbodi O. Thamchaipenet A. Rungchuang J. (2005). Effects of long-term NP-fertilization on abundance and diversity of arbuscular mycorrhizal fungi under a maize cropping system. Plant Soil 270, 371–382. doi: 10.1007/s11104-004-1829-4

[B3] Bindraban P. S. Dimkpa C. O. Pandey R. (2020). Exploring phosphorus fertilizers and fertilization strategies for improved human and environmental health. Biol. Fertil. Soils. 56, 299–317. doi: 10.1007/s00374-019-01430-2

[B4] Camenzind T. Hempel S. Homeier J. Horn S. Velescu A. Wilcke W.. (2014). Nitrogen and phosphorus additions impact arbuscular mycorrhizal abundance and molecular diversity in a tropical montane forest. Global Change Biol. 20, 3646–3659. doi: 10.1111/gcb.12618 24764217

[B5] Campbell B. J. Polson S. W. Hanson T. E. Mack M. C. Schuur E. A. (2010). The effect of nutrient deposition on bacterial communities in Arctic tundra soil. Environ. Microbiol. 12, 1842–1854. doi: 10.1111/j.1462-2920.2010.02189.x 20236166

[B6] Cassman K. G. Peng S. Olk D. C. Ladha J. K. Reichardt W. Dobermann A.. (1998). Opportunities for increased nitrogen-use efficiency from improved resource management in irrigated rice systems. Field Crops Res. 56, 7–39. doi: 10.1016/S0378-4290(97)00140-8

[B7] Chen Y. L. Zhang X. Ye J. S. Han H. Y. Wan S. Q. Chen B. D. (2014). Six-year fertilization modifies the biodiversity of arbuscular mycorrhizal fungi in a temperate steppe in Inner Mongolia. Soil Biol. Biochem. 69, 371–381. doi: 10.1016/j.soilbio.2013.11.020

[B8] Chen Y. P. Rekha P. D. Arun A. B. Shen F. T. Lai W. A. Young C. C. (2006). Phosphate solubilizing bacteria from subtropical soil and their tricalcium phosphate solubilizing abilities. Appl. Soil Ecol. 34, 33–41. doi: 10.1016/j.apsoil.2005.12.002

[B9] Cheng H. Yuan M. Duan Q. Sun R. Shen Y. Yu Q.. (2020). Influence of phosphorus fertilization patterns on the bacterial community in upland farmland. Ind. Crops Products. 155, 112761. doi: 10.1016/j.indcrop.2020.112761

[B10] Chu H. Fierer N. Lauber C. L. Caporaso J. G. Knight R. Grogan P. (2010). Soil bacterial diversity in the Arctic is not fundamentally different from that found in other biomes. Environ. Microbiol. 12 (11), 2998–3006. 20561020 10.1111/j.1462-2920.2010.02277.x

[B11] D’Avolio A. Pensi D. Baietto L. Di Perri G. (2014). Therapeutic drug monitoring of intracellular anti-infective agents. J. Pharm. Biomed. Anal. 101, 183–193. 24768264 10.1016/j.jpba.2014.03.040

[B12] Daniels B. A. (1982). Methods for the recovery and quantitative estimation of propagules from soil. Methods Principles. Mycorrhizal. Res., 29–35.

[B13] Demay J. Ringeval B. Pellerin S. Nesme T. (2023). Half of global agricultural soil phosphorus fertility derived from anthropogenic sources. Nat. Geosci. 16, 69–74. doi: 10.1038/s41561-022-01092-0

[B14] Dincă L. C. Grenni P. Onet C. Onet A. (2022). Fertilization and soil microbial community: a review. Appl. Sci. 12, 1198. doi: 10.3390/app12031198

[B15] Donipati J. Moses S. Rao T. (2023). Development of a manually operated fertilizer applicator with precision metering mechanism for enhanced crop growth and sustainable agriculture. Pharma. Innovation SP-12, 1929–1935. doi: 10.22271/tpi

[B16] Dordas C. (2009). Dry matter, nitrogen and phosphorus accumulation, partitioning and remobilization as affected by N and P fertilization and source-sink relations. Eur. J. Agron. 30, 129–139. doi: 10.1016/j.eja.2008.09.001

[B17] Edgar R. C. (2013). UPARSE: highly accurate OTU sequences from microbial amplicon reads. Nat. Methods 10, 996. doi: 10.1038/nmeth.2604 23955772

[B18] El-Sawah A. M. Abdel-Fattah G. G. Holford P. Korany S. M. Alsherif E. A. AbdElgawad H.. (2023). Funneliformis constrictum modulates polyamine metabolism to enhance tolerance of Zea mays L. @ to salinity. Microbiol. Res. 266, 127254. doi: 10.1016/j.micres.2022.127254 36371871

[B19] Fageria N. K. (2014). Yield and yield components and phosphorus use efficiency of lowland rice genotypes. J. Plant Nutr. 37, 979–989. doi: 10.1080/01904167.2014.888735

[B20] Fageria N. K. Baligar V. C. (2003). Methodology for evaluation of lowland rice genotypes for nitrogen use efficiency. J. Plant Nutr. 26, 1315–1333. doi: 10.1081/PLN-120020373

[B21] Fao F. A. O. S. T. A. T. (2008). Food and agriculture organisation of the United Nations (Rome, Italy: Food and Agriculture Organization of the United Nations), Retrieved on, 15.

[B22] Fierer N. Jackson J. A. Vilgalys R. Jackson R. B. (2005). Assessment of soil microbial community structure by use of taxon-specific quantitative PCR assays. App. Environ. Microbiol. 71 (7), 4117–4120. 10.1128/AEM.71.7.4117-4120.2005 PMC1169028 16000830

[B23] Gosling P. Mead A. Proctor M. Hammond J. P. Bending G. D. (2013). Contrasting arbuscular mycorrhizal communities colonizing different host plants show a similar response to a soil phosphorus concentration gradient. New Phytol. 198, 546–556. doi: 10.1111/nph.12169 23421495 PMC3798118

[B24] Grant C. A. Monreal M. A. Irvine R. B. Mohr R. M. McLaren D. L. Khakbazan M. (2010). Preceding crop and phosphorus fertilization affect cadmium and zinc concentration of flaxseed under conventional and reduced tillage. Plant Soil 333, 337–350. doi: 10.1007/s11104-010-0349-7

[B25] Guo L. Zheng S. Cao C. Li C. (2016). Tillage practices and straw-returning methods affect topsoil bacterial community and organic C under a rice-wheat cropping system in central China. Sci. Rep. 6, 33155. doi: 10.1038/srep33155 27611023 PMC5017303

[B26] Hedley M. J. Stewart J. W. B. Chauhan B. (1982). Changes in inorganic and organic soil phosphorus fractions induced by cultivation practices and by laboratory incubations 1. Soil Sci. Soc. America J. 46, 970–976. doi: 10.2136/sssaj1982.03615995004600050017x

[B27] Jakobsen I. Abbott L. K. Robson A. D. (1992). External hyphae of vesicular—arbuscular mycorrhizal fungi associated with Trifolium subterraneum L. 2. Hyphal transport of 32P over defined distances. New Phytol. 120, 509–516. doi: 10.1111/j.1469-8137.1992.tb01800.x

[B28] Johnson N. C. Wilson G. W. Wilson J. A. Miller R. M. Bowker M. A. (2015). Mycorrhizal phenotypes and the L aw of the M inimum. New Phytol. 205, 1473–1484. doi: 10.1111/nph.13172 25417818

[B29] Lambers H. (2022). Phosphorus acquisition and utilization in plants. Annu. Rev. Plant Biol. 73, 17–42. doi: 10.1146/annurev-arplant-102720-125738 34910587

[B30] Lang M. Christie P. Zhang J. Li X. (2018). Long-term phosphorus application to a maize monoculture influences the soil microbial community and its feedback effects on maize seedling biomass. Appl. Soil Ecol. 128, 12–22. doi: 10.1016/j.apsoil.2018.01.005

[B31] Li H. P. Han Q. Q. Liu Q. M. Gan Y. N. Rensing C. Rivera W. L.. (2023). Roles of phosphate-solubilizing bacteria in mediating soil legacy phosphorus availability. Microbiol. Res. 272, 127375. doi: 10.1016/j.micres.2023.127375 37058784

[B32] Li H. Shen J. Zhang F. Clairotte M. Drevon J. J. Le Cadre E.. (2008). Dynamics of phosphorus fractions in the rhizosphere of common bean (Phaseolus vulgaris L.) and durum wheat (*Triticum turgidum durum* L.) grown in monocropping and intercropping systems. Plant Soil 312, 139–150. doi: 10.1007/s11104-007-9512-1

[B33] Lin X. Feng Y. Zhang H. Chen R. Wang J. Zhang J.. (2012). Long-term balanced fertilization decreases arbuscular mycorrhizal fungal diversity in an arable soil in North China revealed by 454 pyrosequencing. Environ. Sci. Technol. 46, 5764–5771. doi: 10.1021/es3001695 22582875

[B34] Liu X. Sheng H. Jiang S. Yuan Z. Zhang C. Elser J. J. (2016a). Intensification of phosphorus cycling in China since the 1600s. Proc. Natl. Acad. Sci. 113, 2609–2614. doi: 10.1073/pnas.1519554113 26903638 PMC4790974

[B35] Liu Y. Shi G. Mao L. Cheng G. Jiang S. Ma X.. (2012). Direct and indirect influences of 8 yr of nitrogen and phosphorus fertilization on Glomeromycota in an alpine meadow ecosystem. New Phytol. 194, 523–535. doi: 10.1111/j.1469-8137.2012.04050.x 22292929

[B36] Liu W. Zhang Y. Jiang S. Deng Y. Christie P. Murray P. J.. (2016b). Arbuscular mycorrhizal fungi in soil and roots respond differently to phosphorus inputs in an intensively managed calcareous agricultural soil. Sci. Rep. 6, 1–11. doi: 10.1038/srep24902 27102357 PMC4840358

[B37] Lopes A. R. Faria C. Prieto-Fernández Á. Trasar-Cepeda C. Manaia C. M. Nunes O. C. (2011). Comparative study of the microbial diversity of bulk paddy soil of two rice fields subjected to organic and conventional farming. Soil Biol. Biochem. 43, 115–125. doi: 10.1016/j.soilbio.2010.09.021

[B38] Mander C. Wakelin S. Young S. Condron L. O’Callaghan M. (2012). Incidence and diversity of phosphate-solubilising bacteria are linked to phosphorus status in grassland soils. Soil Biol. Biochem. 44, 93–101. doi: 10.1016/j.soilbio.2011.09.009

[B39] Masoni A. Ercoli L. Mariotti M. Arduini I. (2007). Post-anthesis accumulation and remobilization of dry matter, nitrogen and phosphorus in durum wheat as affected by soil type. Eur. J. Agron. 26, 179–186. doi: 10.1016/j.eja.2006.09.006

[B40] Mauchline T. H. Malone J. G. (2017). Life in earth-the root microbiome to the rescue? Curr. Opin. Microbiol. 37, 23–28. doi: 10.1016/j.mib.2017.03.005 28437662

[B41] Mbuthia L. W. Acosta-Martínez V. DeBruyn J. Schaeffer S. Tyler D. Odoi E.. (2015). Long term tillage, cover crop, and fertilization effects on microbial community structure, activity: Implications for soil quality. Soil Biol. Biochem. 89, 24–34. doi: 10.1016/j.soilbio.2015.06.016

[B42] Mei P. P. Gui L. G. Wang P. Huang J. C. Long H. Y. Christie P.. (2012). Maize/faba bean intercropping with rhizobia inoculation enhances productivity and recovery of fertilizer P in a reclaimed desert soil. Field Crops Res. 130, 19–27. doi: 10.1016/j.fcr.2012.02.007

[B43] Nacke H. Thürmer A. Wollherr A. Will C. Hodac L. Herold N.. (2011). Pyrosequencing-based assessment of bacterial community structure along different management types in German forest and grassland soils. PloS One 6, e17000. doi: 10.1371/journal.pone.0017000 21359220 PMC3040199

[B44] Nader A. A. Hauka F. I. A. Afify A. H. El-Sawah A. M. (2024). Drought-Tolerant Bacteria and Arbuscular Mycorrhizal Fungi Mitigate the Detrimental Effects of Drought Stress Induced by Withholding Irrigation at Critical Growth Stages of Soybean (*Glycine max*, L.). Microorganisms. doi: 10.3390/microorganisms12061123 PMC11205826 38930505

[B45] Nelson N. O. Roozeboom K. L. Yeager E. A. Williams J. R. Zerger S. E. Kluitenberg G. J.. (2023). Agronomic and economic implications of cover crop and phosphorus fertilizer management practices for water quality improvement. J Environ Qual. (2023) 52(1):113–125. doi: 10.1002/jeq2.20427 36343334

[B46] Oksanen J. Blanchet F. G. Kindt R. Legendre P. Minchin P. O'Hara B.. (2017). Vegan: Community Ecology Package. Version 2.3-3. Available online at: https://cran.r-project.org/web/packages/vegan/.

[B47] Olsen S. R. (1954). Estimation of available phosphorus in soils by extraction with sodium bicarbonate (No. 939) (Washington, DS, USA: US Dept. of Agriculture).

[B48] Pan L. Cai B. (2023). Phosphate-solubilizing bacteria: advances in their physiology, molecular mechanisms and microbial community effects. Microorganisms. doi: 10.3390/microorganisms11122904 PMC10745930 38138048

[B49] Papakosta D. K. (1994). Phosphorus accumulation and translocation in wheat as affected by cultivar and nitrogen fertilization. J. Agron. Crop Sci. 173 (34), 260–270.

[B50] Peng Y. Duan Y. S. Huo W. G. Xu M. G. Yang X. Y. Wang X. H.. (2021). Soil microbial biomass phosphorus can serve as an index to reflect soil phosphorus fertility. Biol. Fertil. Soils. 57, 657–669. doi: 10.1007/s00374-021-01559-z

[B51] Peng Z. Li C. (2005). Transport and partitioning of phosphorus in wheat as affected by P withdrawal during flag-leaf expansion. Plant Soil 268, 1–11. doi: 10.1007/s11104-004-0297-1

[B52] Powers S. M. Bruulsema T. W. Burt T. P. Chan N. I. Elser J. J. Haygarth P. M.. (2016). Long-term accumulation and transport of anthropogenic phosphorus in three river basins. Nat. Geosci. 9, 353. doi: 10.1038/ngeo2693

[B53] Rathke G. W. Behrens T. Diepenbrock W. (2006). Integrated nitrogen management strategies to improve seed yield, oil content and nitrogen efficiency of winter oilseed rape (Brassica napus L.): a review. Agricult. Ecosyst. Environ. 117, 80–108. doi: 10.1016/j.agee.2006.04.006

[B54] Richardson A. E. Simpson R. J. (2011). Soil microorganisms mediating phosphorus availability update on microbial phosphorus. Plant Physiol. 156, 989–996. doi: 10.1104/pp.111.175448 21606316 PMC3135950

[B55] Rillig M. C. Wright S. F. Shaw M. R. Field C. B. (2002). Artificial climate warming positively affects arbuscular mycorrhizae but decreases soil aggregate water stability in an annual grassland. Oikos 97, 52–58. doi: 10.1034/j.1600-0706.2002.970105.x

[B56] Rousk J. Bååth E. Brookes P. C. Lauber C. L. Lozupone C. Caporaso J. G.. (2010). Soil bacterial and fungal communities across a pH gradient in an arable soil. The ISME J 4 (10), 1340–1351. doi: 10.1038/s41586-022-05220-z 20445636

[B57] Sharma S. B. Sayyed R. Z. Trivedi M. H. Gobi T. A. (2013). Phosphate solubilizing microbes: sustainable approach for managing phosphorus deficiency in agricultural soils. Springerplus 2, 587. doi: 10.1186/2193-1801-2-587 25674415 PMC4320215

[B58] Sharpley A. Tunney H. (2000). Phosphorus research strategies to meet agricultural and environmental challenges of the 21st century. J. Environ. Qual. 29, 176–181. doi: 10.2134/jeq2000.00472425002900010022x

[B59] Shen C. Xiong J. Zhang H. Feng Y. Lin X. Li X.. (2013). Soil pH drives the spatial distribution of bacterial communities along elevation on Changbai Mountain. Soil Biol. Biochem. 57, 204–211. doi: 10.1016/j.soilbio.2012.07.013

[B60] Sheteiwy M. S. El-Sawah A. M. Kobae Y. Basit F. Holford P. Yang H.. (2023). The effects of microbial fertilizers application on growth, yield and some biochemical changes in the leaves and seeds of guar (*Cyamopsis tetragonoloba* L.). Food Res. Int. 172, 113122. doi: 10.1016/j.foodres.2023.113122 37689887

[B61] Smith S. E. Read D. J. (2008). “Arbuscular mycorrhizas,” in Mycorrhizal Symbiosis. Eds. Smith S. E. Read D. J. (Academic Press, London), 11–145.

[B62] Song Y. Yao S. Li X. N. Wang T. Jiang X. Bolan N.. (2023). Soil metabolomics: Deciphering underground metabolic webs in terrestrial ecosystems. Eco-Environment. Health 3, 227–237. doi: 10.1016/j.eehl.2024.03.001 PMC11047296 38680731

[B63] Spohn M. Treichel N. S. Cormann M. Schloter M. Fischer D. (2015). Distribution of phosphatase activity and various bacterial phyla in the rhizosphere of Hordeum vulgare L. depending on P availability. Soil Biol. Biochem. 89, 44–51. doi: 10.1016/j.soilbio.2015.06.018

[B64] Tabatabai M. A. (1982). “Soil enzymes,” in Methods of Soil Analysis. Eds. Miller A. Keeney R. D. (American Society for Agronomy, Madison), 903–947.

[B65] Tan H. Barret M. Mooij M. J. Rice O. Morrissey J. P. Dobson A.. (2013). Long-term phosphorus fertilisation increased the diversity of the total bacterial community and the phoD phosphorus mineraliser group in pasture soils. Biol. Fertil. Soils. 49, 661–672. doi: 10.1007/s00374-012-0755-5

[B66] Tripathi B. M. Stegen J. C. Kim M. Dong K. Adams J. M. Lee Y. K. (2018). Soil pH mediates the balance between stochastic and deterministic assembly of bacteria. ISME. J. 12, 1072. doi: 10.1038/s41396-018-0082-4 29515169 PMC5864241

[B67] Vandamme E. Rose T. Saito K. Jeong K. Wissuwa M. (2016). Integration of P acquisition efficiency, P utilization efficiency and low grain P concentrations into P-efficient rice genotypes for specific target environments. Nutrient. Cycling. Agroecosyst. 104, 413–427. doi: 10.1007/s10705-015-9716-3

[B68] Wang K. Cui K. Liu G. Luo X. Huang J. Nie L.. (2017b). Low straw phosphorus concentration is beneficial for high phosphorus use efficiency for grain production in rice recombinant inbred lines. Field Crops Res. 203, 65–73. doi: 10.1016/j.fcr.2016.12.017

[B69] Wang Q. Garrity G. M. Tiedje J. M. Cole J. R. (2007). Naive Bayesian classifier for rapid assignment of rRNA sequences into the new bacterial taxonomy. Appl. Environ. Microbiol. 73, 5261–5267. doi: 10.1128/AEM.00062-07 17586664 PMC1950982

[B70] Wang C. White P. J. Li C. J. (2017a). Colonization and community structure of arbuscular mycorrhizal fungi in maize roots at different depths in the soil profile respond differently to phosphorus inputs on a long-term experimental site. Mycorrhiza 27, 369–381. doi: 10.1007/s00572-016-0757-5 28039601

[B71] Wang Y. Zhao X. Guo Z. Jia Z. Wang S. Ding K. (2018). Response of soil microbes to a reduction in phosphorus fertilizer in rice-wheat rotation paddy soils with varying soil P levels. Soil Tillage. Res. 181, 127–135. doi: 10.1016/j.still.2018.04.005

[B72] Wang Y. Zhao X. Wang L. Wang Y. Li W. Wang S. Q.. (2015). The regime and P availability of omitting P fertilizer application for rice in rice/wheat rotation in the Taihu Lake Region of southern China. J. Soils. Sediments. 15, 844–853. doi: 10.1007/s11368-014-1047-5

[B73] Williams A. Kane D. A. Ewing P. M. Atwood L. W. Jilling A. Li M. (2016). Soil functional zone management: a vehicle for enhancing production and soil ecosystem services in row-crop agroecosystems. Front. Plant Sci. 7, 65 26904043 10.3389/fpls.2016.00065 PMC4743437

[B74] Xie Y. Niu J. Gan Y. Gao Y. Li A. (2014). Optimizing phosphorus fertilization promotes dry matter accumulation and P remobilization in oilseed flax. Crop Sci. 54, 1729–1736. doi: 10.2135/cropsci2013.10.0672

[B75] Yu X. Keitel C. Dijkstra F. A. (2021). Global analysis of phosphorus fertilizer use efficiency in cereal crops. Global Food Secur. 29, 100545. doi: 10.1016/j.gfs.2021.100545

[B76] Zhao J. Zhang R. Xue C. Xun W. Sun L. Xu Y.. (2014). Pyrosequencing reveals contrasting soil bacterial diversity and community structure of two main winter wheat cropping systems in China. Microbial. Ecol. 67, 443–453. doi: 10.1007/s00248-013-0322-0 24276539

[B77] Zou T. Zhang X. Davidson E. A. (2022). Global trends of cropland phosphorus use and sustainability challenges. Nature 611, 81–87. doi: 10.1038/s41586-022-05220-z 36224391

