# Supplementary material for: Oilseed flax cultivation: optimizing phosphorus use for enhanced growth and soil health
Source: Front Plant Sci. 2024 Sep 11;15:1432875. doi: 10.3389/fpls.2024.1432875 (PMC11422101; doi:10.3389/fpls.2024.1432875)
Supplement: Supplementary file 1 [file Table1.pdf]

Oilseed Flax Cultivation: Optimizing Phosphorus Use for Enhanced Growth and Soil Health

Ning He [a #], Fang Huang [a #], Dingyu Luo [b], Zhiwei Liu [c], Mingming Han [d], Zhigang Zhao [a*] and Xian Sun [b*]

[a] Yichun Key Laboratory of Functional Agriculture and Ecological Environment, Yichun University, Yichun 336000, China;

[b] School of Marine Sciences, Zhuhai Key Laboratory of Marine Bioresources and Environment, Guangdong Provincial Key Laboratory of Marine Resources and Coastal Engineering, Pearl River Estuary Marine Ecosystem Research Station, Ministry of Education, Research Center of Ocean Climate, Sun Yat-Sen University, Southern Marine Science and Engineering Guangdong Laboratory (Zhuhai), Zhuhai 519082, China;

[b] School of Ecology, Sun Yat-sen University, Guangzhou 510275, China;

[d] Biology Program, School of Distance Education, Universiti Sains Malaysia, 11800 Minden, Penang, Malaysia.

# These authors contributed equally to this work.

* Corresponding author e-mail: zhaozg_77@163.com (G. Zhao)

sunx27@mail.sysu.edu.cn (X. Sun)

Table S1 Basic chemical properties of the experiment field.

Time	Organic Matter	Total Nitrogen	Avail. Nitrogen	Avail. Phosphorus	Avail. Potassium
2015	15.22	0.79	67.31	6.85	97.44
2016	14.41	0.67	64.23	6.73	94.75
2017	15.23	0.73	65.77	6.49	93.72

Table S2 Effects of P-fertilizer inputs on different P fractions

P fractionation	Resin P	$NaHCO_3$-Pi	$NaHCO_3$-PO	NaOH-Pi	HCl-Pi	Residual-P	Total-P
P0	1.47c	3.47c	1.78d	4.71c	127.37d	71.32c	201.43c
P40	3.72b	6.73b	4.71c	6.78b	241.57c	90.44b	231.47b
P80	4.85a	8.97b	14.37b	8.44b	276.31b	98.73b	281.31a
P120	5.57a	13.46a	19.73a	11.32a	310.73a	112.43a	297.46a
One-way NOWA	**	***	*	*	**	**	NS

Significant differences among four P-fertilizer inputs within each variable are indicated by dissimilar lowercase letters according to the Duncan test. Additionally, *, **, and *** separately represent $P<0.05$, 0.01, and 0.001, while NS represents not significant.
